# Designing a Mobile Health App for Patients With Dysphagia Following Head and Neck Cancer: A Qualitative Study

**DOI:** 10.2196/rehab.6319

**Published:** 2017-03-24

**Authors:** Gabriela Constantinescu, Irene Loewen, Ben King, Chris Brodt, William Hodgetts, Jana Rieger

**Affiliations:** ^1^ Department of Communication Sciences and Disorders University of Alberta Edmonton, AB Canada; ^2^ Institute for Reconstructive Sciences in Medicine (iRSM) Covenant Health Misericordia Community Hospital Edmonton, AB Canada; ^3^ Department of Industrial Design University of Alberta Edmonton, AB Canada

**Keywords:** app design, dysphagia, games for health, gamification, head and neck cancer, mHealth, mobile health, patient adherence, patient engagement

## Abstract

**Background:**

Adherence to swallowing rehabilitation exercises is important to develop and maintain functional improvement, yet more than half of head and neck cancer (HNC) patients report having difficulty adhering to prescribed regimens. Health apps with game elements have been used in other health domains to motivate and engage patients. Understanding the factors that impact adherence may allow for more effective gamified solutions.

**Objective:**

The aim of our study was to (1) identify self-reported factors that influence adherence to conventional home therapy without a mobile device in HNC patients and (2) identify appealing biofeedback designs that could be used in a health app.

**Methods:**

A total of 10 (4 females) HNC patients (mean=60.1 years) with experience completing home-based rehabilitation programs were recruited. Thematic analysis of semi-structured interviews was used to answer the first objective. Convergent interviews were used to obtain reactions to biofeedback designs.

**Results:**

Facilitators and barriers of adherence to home therapy were described through 6 themes: patient perceptions on outcomes and progress, clinical appointments, cancer treatment, rehabilitation program, personal factors, and connection. App visuals that provide feedback on performance during swallowing exercises should offer an immediate representation of effort relative to a goal. Simple, intuitive graphics were preferred over complex, abstract ones. Continued engagement with the app could be facilitated by tracking progress and by using visuals that build structures with each use.

**Conclusions:**

This is a detailed documentation of the initial steps in designing a health app for a specific patient group. Results revealed the importance of patient engagement in early stages of app development.

## Introduction

### Background

More than half of the patients treated for head and neck cancer (HNC) experience swallowing difficulties also known as dysphagia [1-4]. The inability to swallow safely can have serious consequences on the health and psychosocial well-being of these patients, such as malnourishment, dehydration, aspiration pneumonia, and depression. Although research has shown that individualized, intensive therapy achieves lasting changes to swallowing anatomy and physiology [5], limited clinical resources result in the majority of swallowing therapy prescribed as home programs. Home programs have been reported to have low adherence rates [6] and require clinicians to rely on patient report to measure effectiveness. These limitations render existing approaches to dysphagia treatment inadequate. Technological advancements such as mobile health (mHealth) devices can be combined with existing effective therapies to help address this clinical gap and remotely monitor adherence to treatment regimens.

### mHealth and Swallowing Exercises

The purpose of this study was to obtain patient opinions to inform the design of an mHealth app for swallowing therapy. This app is used together with a wireless mobile device and uses surface electromyography (sEMG) sensors to provide patients with real-time feedback during the exercise. Although it has been recognized that patients prefer more appealing and intuitive displays over signal tracings, the process and research used to select visuals for mHealth apps is rarely reported.

**Figure 1:**
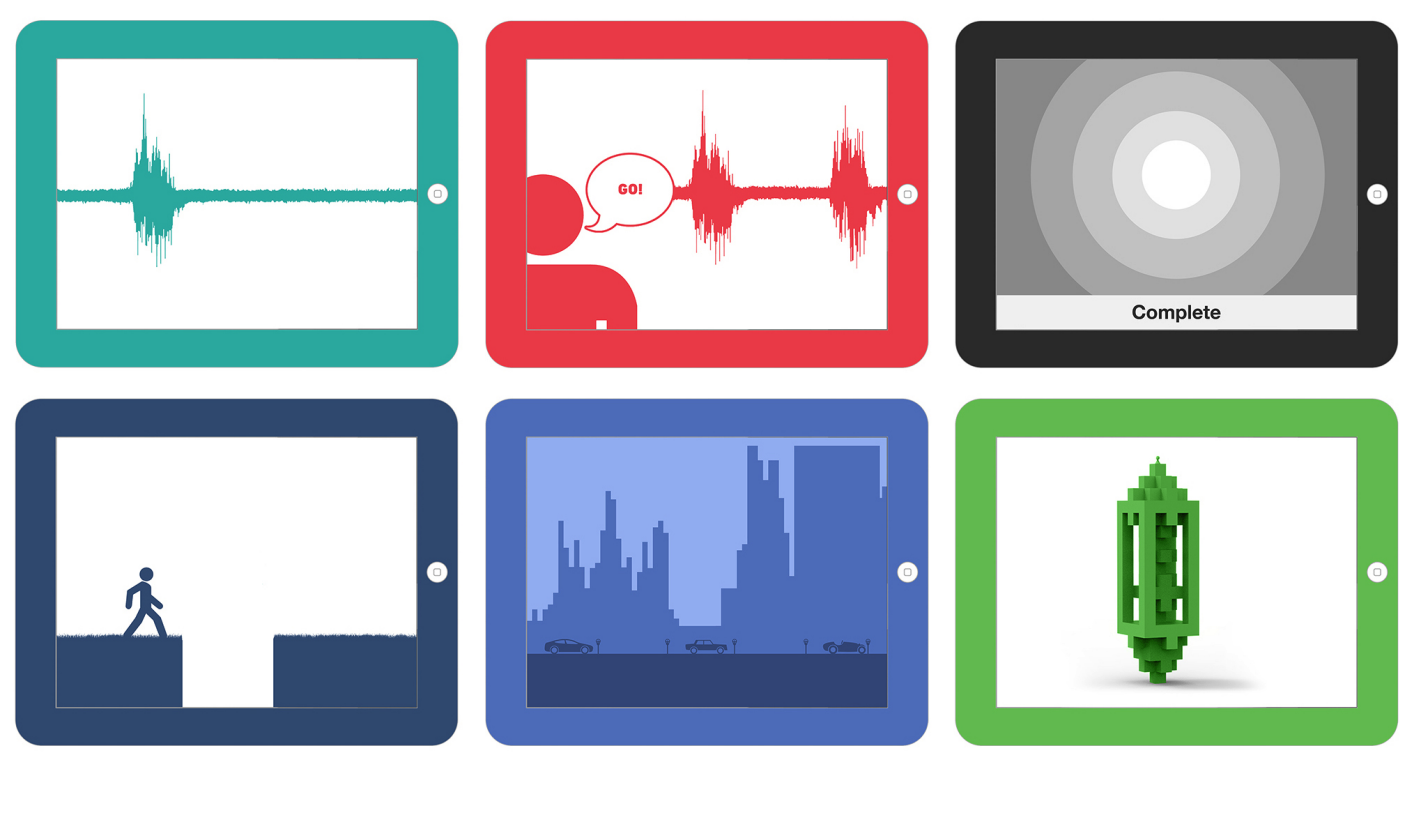
Screenshots of design concepts for visual biofeedback, distinguished across 2 features: the type of visuals (simple, complex, abstract), and the presence or absence of a character. An example for each of the swallow exercises was created for all 6 categories and explained to patients in a video.

Before this study, 6 design concepts for sEMG biofeedback were generated by considering a typical saliva swallow as well as the technique and clinical goals (eg, peak amplitude and duration of contraction) for the 2 swallowing exercises targeted by the app: the effortful swallow and the Mendelsohn maneuver. Two elements were varied in these 6 designs: (1) the level of visual complexity (simple, complex, abstract) and (2) the presence of a character (eg, coach or third person game; Figure 1).

Smeddinck et al (2013) identified visual complexity as an important element to consider in the design of games for health. They surmised from previous work and anecdotal evidence that whereas complex graphics can increase a sense of immersion and motivation in the user, they also can distract patients from their own movements resulting in injury or overexertion [7]. In their study, Smeddinck et al systematically manipulated visual complexity using a taxonomy for common levels of computer graphics ranging from simplified to realistic. The authors found that although visual complexity had no influence on player experience, the older adults perceived greater exertion when realistic visuals were used [7]. The presence of a character (ie, third person games) or a coach is another important element to present to patients as a visual option. The presence of a coach may help patients transition from one-on-one therapy with a clinician to home-based sessions and has been used with other health apps such as My Fitness Coach from Wii. Third person games offer a familiar and predictable game setting and have been successfully used with games for health with pediatric and young adult cancer patients [8].

This study had 2 primary goals, both aimed at contributing to the development of a swallowing therapy app that is engaging to patients with HNC. The first part of patient interviews focused on identifying the determinants of successful adherence to home-based swallowing therapy, information that will be used to select app features (eg, reminders). The second part of the interview focused on obtaining reactions to designs for the visual biofeedback. This aspect of the app was selected because the real-time biofeedback is what participants will rely on as an indicator of correct exercise completion in the absence of a clinician.

### Objectives

The following are our study objectives:

1. What are self-reported determinants for adherence to conventional home therapy (ie, without a mobile device) in patients with dysphagia following treatment for HNC?
2. When shown concepts of visual biofeedback for swallowing therapy exercises that could be used with a mobile device, what are some key design elements that patients with dysphagia feel are important?

Interviewing techniques were selected based on the aim of each objective. Therefore, although each participant took part in a single interview, 2 distinct methods were employed in succession.

## Methods

### Participants

The health research ethics board at the University of Alberta, Edmonton, Alberta, Canada approved this study. Patients with a history of HNC were recruited through tertiary care centers in Edmonton. Participants were included in the study if they reported difficulties with swallowing of any kind and if they had experience with home-based, unsupervised therapy following cancer treatment. This experience was not limited to swallowing exercises, as it is possible that not all participants received home programs for swallowing therapy, but may have had other rehabilitation exercises, such as physiotherapy, prescribed.

### Procedures

Participants were approached either in person or by phone once consent to be contacted by the research team was provided. Participants were booked for an individual appointment, which was split up into 2 parts and videorecorded. Part 1 used a semi-structured approach to explore the facilitators and barriers of adherence to conventional home therapy, without a mobile device. This style of interview allowed for the flexibility to understand individual and unanticipated ideas, but still retained the structure needed for interparticipant comparison [9]. Part 2 of the appointment determined patient preference for visual biofeedback using a convergent interviewing approach. Convergent interviewing is a structured process for explorative research in an emerging field [10,11]. This process has 2 distinguishing features: (1) participants are systematically selected to reflect a wide range of opinions and (2) the process is progressive whereby the initial interview questions, at first unstructured, are used to identify key issues; these findings help focus the questions for subsequent sets of interviews. In this way, converging key issues can be identified [10-12]. Convergent interviews were analyzed in sets of 3; the first 3 interviews (ie, first set) were analyzed for uniting themes, which were then used to guide the interview questions for the subsequent set of 3 appointments. Given that 10 participants were recruited, the first set of convergent interviews comprised 4 participants. An effort was made to ensure that each set of 3 interviews contained participants of different ages and sex. Demographic and past swallowing therapy information was collected at the beginning of the appointment. HNC treatment variables were collected from a chart review. All the participants who were contacted for the study participated.

Interviews were conducted by the first author, a speech-language pathologist with clinical expertise in interviewing this population. As these were her first interviews conducted for research purposes, several pilots were conducted. Recordings took place at 2 locations, each with an identical setup. All participants were told that this study was part of a larger research goal to develop an mHealth device for swallowing therapy with sEMG sensor technology.

### Semi-structured Interviews (Part 1)

Participants were comfortably seated in a room with the interviewer. To explore patient perceived barriers and facilitators to completing conventional swallowing exercises at home, an open-ended question was asked to all participants: “Throughout your cancer treatment, you may have been given some exercises by your speech therapist or your physical therapist. What is your honest opinion about having to do these exercises?” Questions that followed were composed using the Rogers et al theoretical framework for physical activity behavior in patients with HNC [13] as a guide (Multimedia Appendix 1). During the interviews, follow-up questions were used to obtain more in-depth information from participants; as such, no 2 interviews were identical.

The interviews were transcribed verbatim, and identifiers such as names of family, friends, or clinicians were removed [9,14]. Thematic analysis was data-driven and semantic themes (ie, using the surface meaning of data) were sought [9]. Two investigators (GC, IL) coded the transcripts independently, using NVivo for Mac, version 11.1.1 (QSR International Pty Ltd). Lab notes were kept in NVivo and the study binder. Once consensus was reached, transcripts were recoded using the mutually agreed upon set of codes. Codes were grouped into themes and subthemes [15] using Coggle (coggle.it, Cambridge, England).

### Convergent Interviews (Part 2)

During this part of the interview, a second interviewer was called in the room to participate with the first 3 participants. This was done to ensure that questions specific to design were addressed and that design ideas for biofeedback were interpreted correctly for participants (eg, what will happen if the exercise target is unmet in a given design concept). Once the clinician felt comfortable addressing all topics independently, the second interviewer no longer took part. Each participant was introduced to, and asked to try the effortful and the Mendelsohn maneuver swallowing exercises to gain a sense of the effort and focus required to complete them. Next, they were introduced to visual biofeedback and its potential to aid in completing the demonstrated exercises. Participants were presented a short video displaying the 6 distinct visual biofeedback concepts. Patients were then asked a series of questions (Multimedia Appendix 1) to identify distinct visual biofeedback elements of importance to them with respect to swallowing exercises. This approach, like the first part, required broad and open initial questions to encourage interviewees to share as much information as possible without biasing prompts [10]. On occasion, questions were posed again to allow participants to reflect on what had already been shared.

Three groupings of participant appointments were booked. Interviews in the first set were transcribed and analyzed to determine key design themes. These were defined as a topic or element that was brought up by at least two participants in a set of interviews. It did not matter if participants in the set agreed or disagreed on the theme. When an issue was brought up by only 1 interviewee, it was noted, but not regarded as key [10]. Two researchers (GC, CB) independently analyzed the transcripts and identified key design themes through consensus.

**Figure 2:**
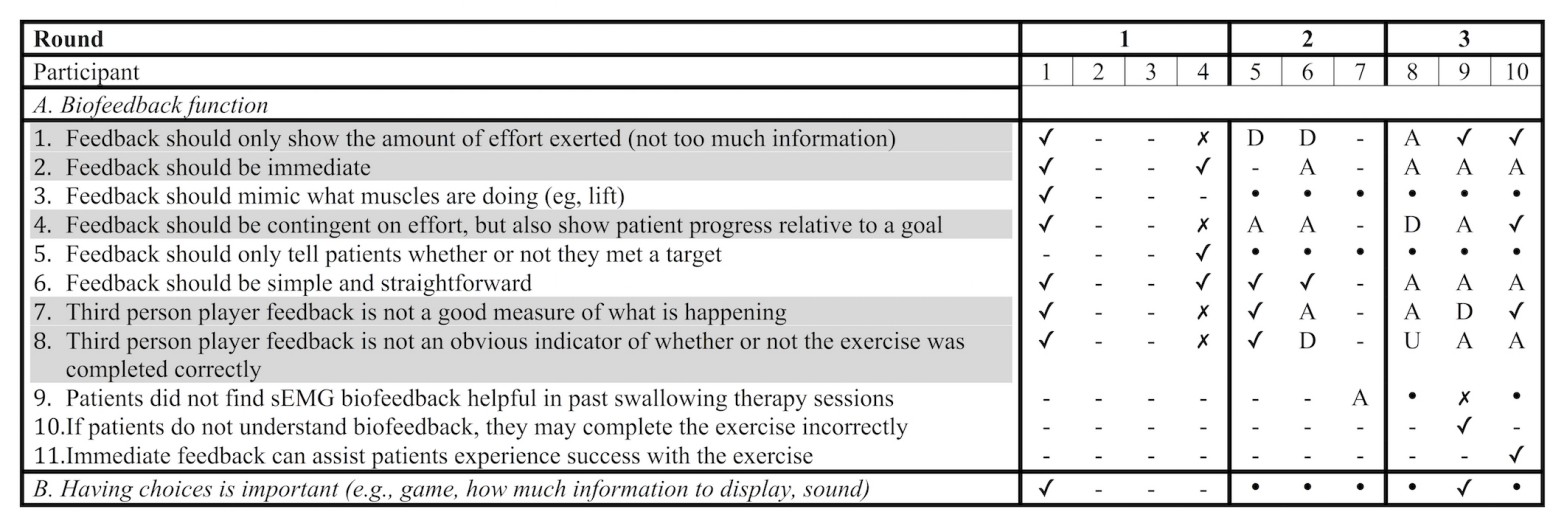
Fragment of notes taken during the analysis of convergent interviews. The following codes were used: (✓) participant agreed with issue; (✗) participant disagreed with issue; (-) participant did not raise this issue, or issue was not probed by clinician; (A) issue actively probed for by interviewer in subsequent set and participant agreed; (D) issue actively probed for by interviewer in subsequent set and participant disagreed; (U) issue actively probed for by interviewer in subsequent set and participant undecided or gave contradicting statements throughout the interview; (.) not a converging theme from previous set and not specifically probed for by interviewer. Highlighted issues were deemed convergent.

In subsequent sets of interviews, the interviewer sought to expand on and to clarify these key design topics. Once new interview questions were generated, the industrial designers and a second clinician vetted them before the start of a new set of interviews. Rao et al (2003) point out that as interview data are collected, new insights may emerge, prompting reexamination of the literature and reshaping ideas for subsequent interviews. If a participant in the second or third group of interviews raised a new topic, it was noted, but not further probed in subsequent discussions unless at least one other interviewee in that set also brought up that topic (Figure 2). Following analysis of all convergent interviews, themes were once again analyzed to determine if they were suitably categorized.

## Results

### Demographics

The study sample comprised a convenience sample of patients visiting the center for various reasons. Descriptive statistics are summarized in Table 1. Although 9 patients complained of dysphagia, only 7 reported having been prescribed swallowing exercises to do at home. One participant reflected mostly on his shoulder rehabilitation exercises, whereas another on his voice therapy. One participant had just begun his radiation therapy at the time of the interview and reported reduced taste sensation. Although this participant had experienced mild pain with swallowing at the time of recruitment, this had resolved. Six participants had prior experience with sEMG as an adjuvant to swallowing therapy in the clinic.

Semi-structured interviews were on average 41 minutes in length (range 19 to 67 minutes), whereas convergent interviews lasted on average 40 minutes (range 27 to 57 minutes). As these 2 interviews addressed different objectives, they will be reported on separately.

**Table 1:** Participant information.

Sex^a^	Age	T-stage	Education	Annual household income (Can $)	Dysphagia history	Past swallowing therapy
Female	45	T2	University	> 80,000	8 months	Yes
Male	64	T1	High school	< 20,000	7 years	No
Male	57	Tx	College	(left blank)	6 months	Yes
Male	66	T1	College	> 80,000	Not applicable	Yes
Female	61	T2	High school	60,000-79,999	5 years	Yes
Female	60	T2	University	> 80,000	8 years	Yes
Male	70	T3	University	(left blank)	5 years	Yes
Female	68	T4	(left blank)	(left blank)	1 year 2 months	Yes
Male	60	T3	High school	< 20,000	16 years 3 months	Yes
Male	50	T2	College	> 80,000	7 years 10 months	Yes

### Semi-structured Interviews (Part 1)

A total of 74 mutually agreed upon set of codes were identified; 5 of these codes were used to mark important information, but were not relevant to the research question (eg, frequency and format of home exercises). Codes were organized into 6 distinct themes: (1) perceptions on outcomes and progress, (2) role of clinical appointments, (3) cancer treatment, (4) rehabilitation program, (5) personal factors, and (6) connection. Facilitators and barriers of adherence to unsupervised home therapy, as explained by these themes, are summarized in Table 2.

The first theme, perceptions on outcomes and progress *,* revealed a potential link in adherence to the gap perceived by patients between their current function and their goal, or their progress toward that goal. Both facilitators and barriers to adherence were evident in this theme. The second theme, role of clinical appointments, included comments on how clinical appointments and clinicians serve to promote adherence. Clinical appointments provided a place for patients to receive education on the anatomy and physiology of a swallow and on how prescribed exercises could improve current function. The use of technology such as biofeedback and modified barium swallow videos facilitated education. These appointments also served as an opportunity to build confidence; patients welcomed reassurance from clinicians if they felt guilty about not completing the full treatment regimen and if they second-guessed their exercise performance. Patients also appreciated clinical appointments as they provided an opportunity to have exercise prescriptions tailored to their needs and abilities. Finally, appointments provided reminders and accountability for doing the exercises. Only facilitators were identified in this theme, although 2 participants brought up a wish for better access.

The third theme, cancer treatment, described various barriers to adherence that relate to surgery, radiation therapy, or chemotherapy. Patients mentioned difficulties with memory and focus as well as a sense of being overwhelmed with information and recommendations. Another perceived barrier was lack of energy or weakness, expressed as either general exhaustion or as rapid muscle fatigue when completing the exercises. Various other side effects mentioned included pain, discomfort, swelling, fibrosis, scarring, postradiation hypothyroidism, and depression. The fourth theme, rehabilitation program, revealed that although there were some facilitators and barriers general to the way the rehabilitation regimen had been set up, some factors also depended on the exercises themselves (eg, novelty, complexity) and some were patient-dependent (eg, time of day when exercises would be completed). Some patients preferred to continue to try new types of exercises and asked peers on social media to share their recommendations, whereas 1 patient reported wanting to wait until a technological solution (ie, prosthetic throat) would exist.

The fifth theme, personal factors, revealed that patients were, at least in this context, generally positive and grateful to be alive. They revealed coping skills through their self-talk and self-compassion, respect for the extent of efforts made by their health care workers, and a wish to help others. Only facilitators to adherence were identified in this theme. The last theme, connection, explained the impact made by a patient’s social context (ie, other patients, friends, family) on adherence and on perceptions of current function. On one hand, interactions with other HNC patients provided support; however, it also facilitated peer comparison of function, a code found in 9 out of the 10 participants in this study. If a patient found his or her function to be better than that of other HNC patients, this made that patient feel good. Although this comparison was not explicitly stated as a facilitator of adherence to home-based treatment, it did influence how patients perceived their current function. This shift in perception may be considered an indirect facilitator or barrier of adherence.

In addition to these themes, it became apparent during the interviews that patient perspectives varied on what home-based swallowing therapy was. When answering interview questions, participants referred to a number of different activities, such as stretches (eg, neck, jaw), maneuvers (eg, head tilt, head turn), and rehabilitation exercises (eg, Mendelsohn maneuver, effortful swallow). Two participants considered swallowing in general as the exercise, making questions on adherence difficult to analyze because these patients felt that they were constantly exercising.

**Table 2:** Summary of facilitators and barriers to adherence identified in each theme.

Theme	Factor	Sample quote
**Theme 1: Perceptions on outcomes and progress**			
**Facilitators**	Perceived regression in function or fear of poor outcomes	“I need to work harder at it. And, because, I’ve already been pretty sick, I don’t want to get sick again.”
	Perceived benefit as a result of the exercise	“I did stick with it because I went, ‘Wow, I’d do this.’ Any improvement in swallowing, being able to maybe eat a little faster cuz it’s going down quicker, I want. I really want it.”
**Barriers**	No swallowing problem or restored function	“I told myself, oh I’m in the clear!”
	Perceived little or no progress	“I don’t see any more progress, I’m not doing this anymore.”
	Unrealistic postcancer treatment outcome expectations	“(...) you realize okay well this is gonna take time.”
	Pessimistic adjustment in outcome expectations	“I just resigned myself to the fact that I don’t think my situation is really gonna change.”
**Theme 2: Role of clinical appointments**			
**Facilitators**	Education	“Now, now I see where you-, what you’re getting at, when you invent these exercises.”
	Building confidence	“I was always second-guessing really my technique. So I found the technique a little bit difficult to actually maintain. Um, especially after (...) I would leave the in-house session and try to do them at home.”
	Tailored prescriptions	“But she said if it’s too difficult and you find an issue then just at least continue on with the other ones. Just don’t stop”
	Accountability	“(...) you slide into bad habits pretty fast. If you’re not constantly monitored.”
**Barrier**	Access	“So so if I was doing something wrong, I didn’t have the feedback to tell me try this or try that. I had to wait till my next appointment.”
**Theme 3: Cancer treatment**			
**Barriers**	Memory and focus	“I’d get home and you’d hand it to me, like do this, this and this, and I’d go, ‘Well that’s so simple’ Good God. And I’d get home and go (face palm) ‘What, what (...) oh man, I don’t remember, I don’t know what this means, and I’m not gonna phone because this is grade 3 instructions’ know what I mean?”
	Sense of overwhelm with information and recommendations	“(...) this type of cancer is very complex in its requirements for support and therapy, yeah, some days, it’s just like whoa, it’s a lot to keep on track, I can’t keep it all up.”
	Low energy and fatigue	“So sometimes all I had time for or energy in the day was a 1 hour visit with somebody. Maybe half an hour only. And then exercises, even eating sometimes would fall off because I wanted to go nap and sleep.”
	Other side effects	“You’re tired. You’re tired of choking. You’re miserable. You’re isolated. You can’t communicate as it is except by writing a lot of places. Like for months. After the radiation burns your throat and that, it makes it harder to swallow, your throat’s raw. For so many reasons that make it easy not to, to swallow. And to take the food, there’s just an endless list of reasons why you can say, ‘Well, it’s too hard!’”
**Theme 4: Rehabilitation program**			
**Facilitators**	General: tracking progress, providing reminders, routine, setting goals	“So then I was tracking my swallow exercises at home, which, yeah, helped, I think. Helped to motivate me, to remind me that those were really critical. And helped me to also track how was how well I was doing.”
	Patient-specific: adjusting the practice environment, customizing the exercise schedule	“At first, I’d get up in the morning and do them, kind of when I did my meds and stuff and try and get rid of all that at the same time.”
	Exercise-specific: novel, interesting, easy, tackle multiple goals at once	“(...) but some of the ones were very unique, so there (were) more complex ones where you held (...) your breath. I thought, ‘Oh, actually this is kind of cool’ So it was kind of intriguing for a while.”
**Barriers**	General: no structure, distractions, length of time in rehabilitation program	“(...) But it’s not official, it’s not regimented, it’s not programmed (...)”
	Exercise-specific: too complex or difficult, feeling self-conscious, misinterpreting other activities as exercise	“(...) but after a while the complex ones fell off rather quickly” “So there is an embarrassment factor that you have to get over. But I just go down into in my room in the basement and sortta, I guess isolate myself a lot to do certain exercises.”
**Theme 5: Personal factors**			
**Facilitators**	Positive and grateful	“But then after I started feeling better again, then I thought, ‘Well, the rest of me is getting better, this part might as well come along too’ so, I kind of got back into doing them a little more.”
	Coping, through self-talk and self-compassion	“I would think, ‘Just stop, stop whining, get get up and get better’”. “I would forgive myself that day. And then I would (unintelligible) tomorrow.”
	Sense of personal obligation to health care workers involved in extended treatment	“The thing is to (...) keep it in your mind that the surgeons and the therapists and the nurses and the whoever are the ones that are the reason why you’re here. And you owe it to them and to yourself to, (unintelligible) and to be strong (...).”
	Wish to become a role model or helper	“I think more like, I want to be a role model for my friends. Yeah. I want to show them that if you put your mind to it, you can do it.”
**Theme 6: Connection**			
**(Potential indirect)** **facilitator**	Patient perceives his or her function to be better than that of peers	“It’s not fair, but then there’s others where, like there’s for example the guy that can only eat cream of wheat, I’m going ‘Wow, I’m miles ahead of him!’”
**(Potential indirect)** **barrier**	Patient perceives his or her function to be worse than that of peers	“(...) and it got really depressing, because all these people they would be put on the peg, taken off the peg, off they go. New norm! (...) and they would come in and, ‘Today I ate half a hamburger!’ Well, I ate my first half of hamburger the other day. And this was within 3 months of their treatment (...).”

### Convergent Interviews (Part 2)

A total of 84 issues and 11 preliminary themes were found across all 10 interviews. Of these, 21 were found to be convergent (Table 3). These topics were first explored for level of agreement. All participants who had an opportunity to discuss the following issues agreed that biofeedback should be immediate, simple, and straightforward; noting improvement over time is important and builds confidence; competition with oneself is preferred over competition with peers. Most participants (5 or more) agreed that: feedback should be contingent on effort, but also show user progress relative to a goal; having a third person character is not a good measure of what is happening during the swallow exercise; education is important for uptake and adherence; tracking progress over time is important; and visuals where structures are built over time are engaging. Most participants (5 or more) disagreed with issues raised by some of the participants in the first set of interviews, namely that: visuals with a medical look, such as raw signal, are unappealing; progress graphs are difficult to interpret; completing all assigned swallow trials is important; and that they felt concern for a third person character in the game (ie, did not want character to get hurt if the swallow exercise was not completed well). A split in opinion was noted for the following issues: feedback should only show amount of effort (ie, not overwhelm the user with too much information), that the third person character feedback does not make it obvious if the exercise was completed correctly, that the third person player game is engaging, that more complex visuals are better than simplistic ones, that built-in reminders are beneficial, and finally that failure motivates one to keep trying.

**Table 3:** Convergent themes.

Key issue	Agreed	Disagreed	Undecided or not addressed
Feedback should only show amount of effort (not too much information)	4	3	3
Feedback should be immediate	6	0	4
Feedback should be contingent on effort, but also show progress relative to goal	5	2	3
Feedback should be simple and straightforward	7	0	3
Third person player feedback is not a good measure of what is happening	5	2	3
Third person player feedback does not make it obvious if user completed exercise correctly	4	2	4
Education is important to get patients to do the exercises	6	1	3
Visuals that look medical do not look good (eg, graphs)	2	5	3
Visuals that are more complex are better that those that are too simple	4	4	3
Graphs are difficult to interpret	1	5	4
Artistic creations using biofeedback were nice, but too soft and boring	3	0	7
Completing the number of swallow trials is important	3	5	2
Built-in reminders are beneficial; patients have a lot of time demands	2	2	6
Failure motivates users to keep trying again and work harder	4	3	3
Improvement over time is important; building confidence in swallowing ability	6	0	4
Building structures over time is engaging	5	2	3
Concern expressed for third person player in the game	1	7	2
Third person player game is engaging	3	4	3
Tracking progress over time is important	8	1	1
Tracking progress should include a baseline	3	0	7
Competition with self is better than that with others	5	0	5

## Discussion

### Principal Findings

This study obtained detailed patient feedback on past experiences with home programs and on preferences for app visuals, findings that may generalize to other apps for HNC patients, and apps that use visual biofeedback. The study also offers a detailed documentation of our approach to designing a mobile swallowing therapy app, a methodology that may be applied when developing for other patient groups.

The exploration of determinants for adherence to home therapy revealed a number of elements that could be incorporated in future mHealth apps for swallowing therapy. First, aside from an objective approach to documenting adherence, mHealth apps would provide an opportunity for clinician remote monitoring. Fluctuations in adherence or nonadherence could alert clinicians so that they may target those patients who struggle most. Adjustments to the therapy regimen could be made remotely or in conversation with the patient, retaining an individualized quality to the therapy. For example, this is an existing feature of SwallowSTRONG, an mHealth device and app for tongue strengthening exercises (Swallow Solutions, LLC, Madison, WI). Finally, remote monitoring also provides an avenue for accountability to a clinician.

Second, apps may address any existing or anticipated gaps in access to swallowing therapy or educational information. A mobile device also provides an opportunity for HNC patients to complete exercises during high-energy periods in the day or to customize exercise programs according to medication schedule, rather than to clinician availability.

Third, mHealth devices and apps for swallowing therapy can furthermore address adherence by providing education, instructions, and biofeedback. The app could include educational screens highlighting the importance of regular exercise, and the expected impact that specific exercises are expected to have on swallow physiology. Education on how progress may change throughout the course of cancer treatment also may be important, as some patients reported neglecting their exercises when function appeared to improve. Information that can be accessed multiple times, at the user’s convenience, should address concerns raised around the shame of asking for help. The app could track progress over time and use that information to demonstrate incremental improvements.

Two additional important elements that should be considered in a swallowing therapy mHealth app relate to biofeedback and social engagement. First, the biofeedback should be accurate and precise enough so that appropriate techniques are reinforced and frustration is minimized. Second, although leaderboards and status shares are important elements in many other health apps, our findings suggest that these are not recommended for swallowing therapy in HNC patients. Peer-to-peer comparison of performance may result in poor self-efficacy and lead to depression; however, social engagement in the app may take on other forms such as an anonymous patient-to-patient exchange of motivational messages.

Finally, some aspects of adherence appeared to be best mediated during clinical appointments. These included forming realistic expectations, building hope, and managing treatment side effects such as pain.

With respect to the development of our app, the following design recommendations were made once converging themes were synthesized. Visual biofeedback should be immediate and relative to the level of muscle activity detected. It should be represented simply so that it is easily understood. Since mixed opinions occurred with respect to displaying a reference target during each trial, perhaps this visual can be set to on or off based on user preferences.

With respect to visuals in the app, there was no real or perceived aversion to the raw signal. Whereas the participants agreed that it looked medical, most preferred it because they found it easy to interpret. An interesting finding was that typical game-play (ie, third person character jumping or ducking over obstacles) was not meaningful to the patients in this study and should be avoided for swallowing therapy apps. However, the act of constructing something over time was deemed engaging and even more entertaining than simpler visuals. When biofeedback was represented through expanding shapes and colors, participants felt that the visuals were too soft and uninteresting. Furthermore, irrespective of the visual theme, failure should be presented in a sensitive way. Whereas a few participants felt that failure in the app would be a strong motivator (eg, character falls down a cliff if target is not met), the majority of participants shared that failing in the game would be upsetting: “I would feel defeated. Like oh yeah, don’t even know how to do this.” Finally, tracking improvements over time within the game have the potential to build confidence with the user’s swallowing ability outside of the app.

With respect to app features, participants agreed that education was important, particularly to build an understanding on the importance of completing all trials with maximum effort. Connecting with other HNC patients in the app for the purpose of competition should be avoided. Built-in reminders may help some users, but could be postponed to later app versions as some participants stated that they would not use this feature.

### Limitations

This study consisted of a convenience sample of 10 participants recruited over a period of 6 months. Since these interviews were conducted to inform the design of an app, it is possible that data saturation was not achieved. A time frame of 6 months was deemed a reasonable delay in the development of our mHealth app in order to engage end-users early. Furthermore, although the sample size was small, it was heterogeneous enough (eg, in duration of dysphagia, length of time from cancer treatment, and level of adherence to swallowing exercises) to represent most types of patients using the future mHealth app. In addition, 3 of the 10 participants reported no prior experience with home-based swallowing therapy and had to reflect on other types of rehabilitation exercises. Therefore, the reader is cautioned when interpreting these findings, as the themes identified here may not generalize to all HNC patients or swallowing apps.

Additional limitations include self-selection and recall bias. Two participants were noted to wear a FitBit and 1 participant wore a smartwatch; participants varied in their experience with dysphagia (6 months to 16 years). In addition, we were unable to quantify the strength of a participant’s opinion. For example, how does one distinguish between a participant who has a preference, but not a strong one, and someone who may not complete the exercise program at all if a particular design were selected?

Additional details on the study were compiled with the assistance of the consolidated criteria for reporting qualitative research (COREQ) checklist [16] and are summarized here to assist readers in assessing the level of bias present in this work. The interviewer (GC) and the second coder in part 1 (IL) are female, both with clinical experience in HNC; the industrial designers who assisted with part 2 (BK and CB) are both male. Although the interviewer had prior expertise conducting clinical interviews, this was her first time doing so in a research study. The researchers could not approach patients directly for study recruitment until consent to be contacted by the research team was provided. Therefore, it is unknown how many patients were approached, but declined to be contacted. A prior relationship existed with some patients as the primary interviewer also worked as a clinician. Furthermore, participants did not provide feedback on the transcript accuracy or findings.

### Conclusions

The collection of patient perspectives is an important step in the development of mHealth technologies for a patient population that has not been extensively targeted by this industry. Although a laborious process, the themes identified in this study informed how mHealth apps could be used as an adjuvant to home rehabilitation following treatment for head and neck cancer. This approach also revealed that visuals that appeal to the development team, such a complex graphics with game elements, might not necessarily be intuitive to users.

## Appendix

Multimedia Appendix 1 Semi-structured interview questions.

## Abbreviations

HNC: head and neck cancer
mHealth: mobile health
sEMG: surface electromyography

## References

[1] Arrese LC, Lazarus CL (2013). Special groups: head and neck cancer. Otolaryngol Clin North Am.

[2] Raber-Durlacher JE, Brennan MT, Verdonck-de Leeuw IM, Gibson RJ, Eilers JG, Waltimo T, Bots CP, Michelet M, Sollecito TP, Rouleau TS, Sewnaik A, Bensadoun R, Fliedner MC, Silverman S, Spijkervet FK, Dysphagia Section‚ Oral Care Study Group‚ Multinational Association of Supportive Care in Cancer (MASCC)/International Society of Oral Oncology (ISOO) (2012). Swallowing dysfunction in cancer patients. Support Care Cancer.

[3] Langerman A, Maccracken E, Kasza K, Haraf DJ, Vokes EE, Stenson KM (2007). Aspiration in chemoradiated patients with head and neck cancer. Arch Otolaryngol Head Neck Surg.

[4] Szczesniak MM, Maclean J, Zhang T, Graham PH, Cook IJ (2014). Persistent dysphagia after head and neck radiotherapy: a common and under-reported complication with significant effect on non-cancer-related mortality. Clin Oncol (R Coll Radiol).

[5] Burkhead LM, Sapienza CM, Rosenbek JC (2007). Strength-training exercise in dysphagia rehabilitation: principles, procedures, and directions for future research. Dysphagia.

[6] Shinn EH, Basen-Engquist K, Baum G, Steen S, Bauman RF, Morrison W, Garden AS, Sheil C, Kilgore K, Hutcheson KA, Barringer D, Yuan Y, Lewin JS (2013). Adherence to preventive exercises and self-reported swallowing outcomes in post-radiation head and neck cancer patients. Head Neck.

[7] Smeddinck J, Gerling KM, Tiemkeo S (2013). Visual Complexity, Player Experience, Performance and Physical Exertion in Motion - Based Games for Older Adults. http://hci.usask.ca/uploads/313-ASSETS-VisualComplexity-camreadyFINAL.pdf.

[8] Kato PM, Cole SW, Bradlyn AS, Pollock BH (2008). A video game improves behavioral outcomes in adolescents and young adults with cancer: a randomized trial. Pediatrics.

[9] Braun V, Clarke V (2006). Using thematic analysis in psychology. Qual Res Psychol.

[10] Jepsen DM, Rodwell JJ (2008). Convergent interviewing: a qualitative diagnostic technique for researchers. Management Research News.

[11] Williams W, Lewis D (2005). Convergent interviewing: a tool for strategic investigation. Strat Change.

[12] Rao S, Perry C (2003). Convergent interviewing to build a theory in under‐researched areas: principles and an example investigation of Internet usage in inter‐firm relationships. Qualitative Mrkt Res.

[13] Rogers LQ, Courneya KS, Robbins KT, Malone J, Seiz A, Koch L, Rao K (2008). Physical activity correlates and barriers in head and neck cancer patients. Support Care Cancer.

[14] Cobley CS, Fisher RJ, Chouliara N, Kerr M, Walker MF (2013). A qualitative study exploring patients' and carers' experiences of Early Supported Discharge services after stroke. Clin Rehabil.

[15] Guba EG (1978). Toward a Methodology of Naturalistic Inquiry in Educational Evaluation.

[16] Tong A, Sainsbury P, Craig J (2007). Consolidated criteria for reporting qualitative research (COREQ): a 32-item checklist for interviews and focus groups. Int J Qual Health Care.

