# Supplementary material for: Designing a Mobile Health App for Patients With Dysphagia Following Head and Neck Cancer: A Qualitative Study
Source: JMIR Rehabil Assist Technol. 2017 Mar 24;4(1):e3. doi: 10.2196/rehab.6319 (PMC5454563; doi:10.2196/rehab.6319)
Supplement: Multimedia Appendix 1 [file rehab_v4i1e3_app1.pdf]

Throughout your cancer treatment, you may have been given some exercises by your speech therapist or your physical therapist. What is your honest opinion about having to do these exercises?

Probe: How often did you complete them? What were some of the factors that led you to completing/ not completing your exercises?

Perceived facilitators and barriers

- What do you feel are some things/ factors that help you complete your exercises?
- What do you feel are some things/ factors that keep you from completing your exercises?

Enjoyment

- What types of things help you enjoy your exercises?
- What things make you not enjoy your exercises?

Social support

- What social supports (family, friends) help your complete your exercises?
- What social factors (family, friends, family dynamics) prevent you from completing your exercises?

Role models

- What are some role models (e.g., past patients, friends or family who have gone through something similar) help you complete your exercises?
- What are some things that these role models do, or any things about them, that impede you from completing your exercises?

Depression

- When you’re not having the best of days, what do you do or tell yourself to get yourself to do your exercises anyway?
- In those situations, what helps the most in getting you to do the exercises?

Symptoms

12) What sort of symptoms impede your from doing your exercises?

- When you feel those symptoms, what do you do anything or tell yourself something to get you to stick to your exercises?
- In those situations, what helps the most in getting you to do the exercises?

Additional questions:

13) Do you feel that you knew how to complete the exercises correctly? (i.e., was the exercise demonstrated only once, did you have the opportunity to demonstrate your ability to do the exercise accurately before you were sent home to do them?

14) Did you perceive any change, positive or negative, as a result of the exercises?

Convergent Interview – Questions for Set 1

1) What did you think about the concepts we came up with?
2) What do you think about looking at one of those concepts while doing one of the exercises we practiced earlier?
3) What type of concept would make the exercise more exciting for you?
4) What type of concept would you find most engaging?
5) What type of concept would make it easier for you to do the exercise?
6) What would be most important to you in a feedback like that?
7) What kind of frame of mind do you prefer to be in when doing these exercises? (prompt: When you’re doing a hobby, do you prefer to do it with others, do you like to be alone, do you like to be focused, relaxed?
